# Setting-Specific and Symptom-Specific Association between Secondhand Smoke Exposure and Depressive Symptoms

**DOI:** 10.3390/ijerph16071249

**Published:** 2019-04-08

**Authors:** Xiaohua Ye, Jingya Huang, Liang Xia, Xiaojun Xu, Xiao Gong, Yanjun Xu

**Affiliations:** 1 School of Public Health, Guangdong Pharmaceutical University, Guangzhou 510310, China; jingyi4597@hotmail.com (J.H.); x.gong@foxmail.com (X.G.); 2 Institute of Chronic Noncommunicable Disease and Control, Guangdong Provincial Center for Disease Control and Prevention, Guangzhou 511430, China; xialiangjsxz@126.com (L.X.); xu-yd@163.com (X.X.)

**Keywords:** secondhand smoke, depressive symptoms, influencing factors

## Abstract

Few studies have focused on the potential relationship between secondhand smoke (SHS) exposure and depressive symptoms. This study aimed to explore the potential association between SHS exposure and depressive symptoms and differentiate this association in setting-specific exposure and symptom-specific outcomes. A cross-sectional study was conducted in Guangdong province of China from September to December 2010 using a multistage sampling method to randomly sample adults aged 18 years and older. SHS exposure was defined as inhalation by non-smokers of the smoke exhaled from smokers for at least 1 day a week in the past 30 days. Depressive symptoms were measured using the nine-item Patient Health Questionnaire. The zero-inflate negative binomial regression models were used to explore the associations between SHS exposure and depressive symptoms. A total of 2771 non-smokers were included in this study, with mean age of 49.6 ± 14.0 years and 70.3% of females. The prevalence of depressive symptoms was significantly higher in participants with SHS exposure than in those without exposure (incidence rate ratio (IRR) = 1.32, 95% confidence interval (CI) 1.16–1.51), and there were similar positive associations for SHS exposure in medical facilities (IRR = 1.37, 95% CI 1.17–1.61) and in schools (IRR = 1.46, 95% CI 1.20–1.77). Notably, there was a monotonically increasing dose-response relationship between frequency of SHS exposure and depressive symptoms. When differentiating this relationship by the dimensions of depressive symptoms, there were similar dose-response relationships for cognitive-affective and somatic symptoms. When differentiating this relationship by sex, only females showed a significant dose-response relationship. Our findings suggest dose-response relationships between SHS exposure and depressive symptoms in sex-specific and symptom-specific manners. Future longitudinal studies are needed to establish the biological mechanisms of the impact of SHS exposure.

## 1. Introduction

It is well established that there is no risk-free level of exposure to secondhand smoke (SHS) [1]. In a retrospective analysis of worldwide burden of disease from SHS exposure, 40% of children, 33% of male non-smokers, and 35% of female non-smokers were exposed to SHS, which caused 603,000 deaths (about 1.0% of worldwide mortality) [2]. In China, 72.4% of non-smoking adults were exposed to SHS and 38.0% of adults had regular SHS exposure. Therefore, SHS constitutes a substantial public health threat [3]. In addition, the latest adult survey in Guangzhou, China, revealed that SHS exposure was remarkably high in places with partial smoking ban (with designated smoking rooms) before (89.5%) and after (87.4%) the implementation of Guangzhou smoke-free legislation, which may be due to poor compliance with the law [4]. Although, so far, no national smoke-free law exists in China, Guangzhou is the first city in Guangdong province that implemented smoke-free legislation on 1 September 2010, with a full smoking ban (100% smoke-free) in medical facilities/schools/transport vehicles and a partial smoking ban in restaurants. No smoke-free legislation has been implemented in other cities of Guangdong province (e.g., Yunfu, Shanwei, Meizhou, Zhaoqing, and Shaoguan).

Depressive disorders are the most common mental problems, occurring as early as at three years of age and across all world regions [5,6]. Previous global burden of disease studies revealed that depressive disorders were a leading cause of burden, suggesting depression as a global health priority [7]. With the rapid development of economy, depression disorder is not only a public health issue but also a socioeconomic problem and is attracting more and more attention in China. The latest Chinese epidemiological study revealed that the prevalence rates of depressive symptoms were high in 34.7% of the non-Dibao population and in 50.0% of the Dibao population [8]. In addition, a recent systematic review indicated that prevalence rates of depression or depressive disorder among Chinese children were reported to range from 12.1% to 51.4%, suggesting the need to develop more effective approaches for prevention and management of depression [9]. 

Strong epidemiological evidence has demonstrated that smoking is strongly associated with depression or major depression [10,11]. In addition, the latest systematic review has revealed that stopping smoking is associated with improvements in depression and psychological quality of life [12]. However, the potential relationship between SHS exposure and depressive disorders has not been well established. Although there is increasing evidence for this association, the current findings are inconsistent [13,14,15,16,17,18,19,20]. For example, SHS exposure was significantly associated with increased risk of depressive symptoms in some studies [15,16], but non-significant relations were observed in other studies [14]. These inconsistent results could be due to differences in measurement methods for SHS and depressive symptoms, study design, and populations investigated. In addition, most studies mainly focused on exploring this association for workplace and household SHS exposure [15,16], and few epidemiologic studies have differentiated this association in setting-specific SHS exposure to make the sources of exposure clearer. Although women are more susceptible to SHS-related factors [21,22], and potential biological and behavioral mechanisms have revealed different effects of SHS on cognitive–affective and somatic symptoms [23,24,25,26], few studies have stratified their results by sex and by dimensions of depressive symptoms to make the potential associations clearer. Moreover, it remains unclear whether there are dose-response relationships between SHS exposure and depressive symptoms. Therefore, this study aimed to explore the potential dose-response relationship between SHS exposure and depressive symptoms among healthy adults and also differentiate this association in setting-specific exposure and symptom-specific outcomes.

## 2. Methods

### 2.1. Study Design and Sampling

The study was part of the 2010 China Chronic Disease and Risk Factor Surveillance organized by Guangdong Provincial Center for Disease Control and Prevention. All interviewers in each area were trained to ensure that the survey was carried out according to the protocol and that operation procedures were identical across all areas. The details of the study design and quality control have been described elsewhere [27,28]. Briefly, this cross-sectional study was conducted in Guangdong province of China from September to December 2010. The target population was adults aged 18 years and older. Multistage stratified cluster sampling was used to obtain a representative sample of Guangdong adults. First, six cities (Guangzhou, Yunfu, Shanwei, Meizhou, Zhaoqing, and Shaoguan) were randomly sampled from the 21 cities in Guangdong Province. Second, four townships were randomly selected from each sampled city by using the method of probability proportional to size. Third, three villages or residential areas were then selected from each chosen township using the method of probability proportional to size. Fourth, a residential group (at least 50 families) was selected from each selected village or residential area using the simple random sampling method. Finally, an individual aged 18 years or older was selected in each family by means of a Kish grid. Kish grid is a method for selecting members within a household to be interviewed, and this technique is devised so that all individuals in a household have an equal chance of selection. After obtaining informed consent, eligible participants were selected to complete a face-to-face survey. In this study, only non-smoking adults were included. Thus, data were excluded from those who were unwilling to participate in this survey, those who were smokers, and those who did not complete all the questions used in this study. This resulted in a sample of 2771 non-smokers.

The present study was approved by the Ethics Committee of the Chinese Center for Disease Control and Prevention and was performed in accordance with the approved guidelines (No. 201010).

### 2.2. Depressive Symptoms

The main outcome variable was self-reported depressive symptoms assessed by a Chinese version of the nine-item Patient Health Questionnaire (PHQ-9). Previous research has shown that a PHQ-9 score ≥10 had a sensitivity of 88% and a specificity of 88% for major depression, the area under the curve in diagnosing major depression was 0.95, and there was a strong association between increasing PHQ-9 scores and worsening function, suggesting good construct and criterion validity [29]. Participants were asked for the frequency of occurrence for each symptom on a four-point Likert scale (0: not at all; 1: several days but less than half the days; 2: more than half the days; and 3: nearly every day) in the past two weeks. The aggregate scores of PHQ-9 range from 0 to 27, and higher scores indicate higher levels of depressive symptoms. In addition, on the basis of the factor model for PHQ-9, depressive symptoms were divided into two different dimensions, including cognitive–affective symptoms (item 1, little interest or pleasure in doing things; item 2, feeling down, depressed, or hopeless; item 6, feeling bad about yourself, or that you are a failure, or have let yourself or your family down; item 7, trouble concentrating on things, such as reading a newspaper or watching television; and item 9, thoughts that you would be better off dead, or thoughts of hurting yourself in some way) and somatic symptoms (item 3, trouble falling or staying asleep, or sleeping too much; item 4, feeling tired or having little energy; item 5, poor appetite or overeating; and item 8, moving or speaking so slowly that other people could have noticed, or so fidgety or restless that you have been moving a lot more than usual), which is consistent with a previous study [30]. Cronbach’s alpha for the PHQ-9 in this study was 0.786, suggesting a good internal consistency of the questionnaire.

### 2.3. SHS Exposure

The main independent variable was self-reported SHS exposure. To ascertain the SHS status in general, participants were asked the following question: “During the past 30 days, how many days a week have you breathed in smoke (including tobacco or e-cigarettes) exhaled from smokers in any places?”. SHS exposure in general was defined as inhalation by non-smokers of smoke for at least 1 day a week (1 day means that in that day there are smokers smoking up to 15 min or longer) in the past 30 days [31]. Frequency of SHS exposure in general was reported as a continuous variable (days/week) and was also categorized into two groups (no or yes). Notably, SHS exposure in general was referred to total exposure from anywhere including at work or at home. To ascertain the SHS status in different venues (including indoor medical facilities, indoor and outdoor primary/secondary schools, indoor restaurants, and indoor transport vehicles), the participants were asked a series of questions on whether they had been in any public places in the past 30 days and on whether they had been exposed to SHS in those places. SHS exposure in different venues is a binary variable (no or yes) with no information on frequency of exposure. To ascertain the smoking status, the participants were asked the following questions: “Do you smoke 100 cigarettes or more in your lifetime?” and “Do you currently smoke?”, and those responding “no” to both questions were defined as non-smokers.

### 2.4. Other Influencing Factors

Other influencing factors including potential covariates and confounders were chosen a priori on the basis of a literature review. Other influencing factors in this study included demographic characteristics (age and sex), socioeconomic status (education and per capita family income), physical injury, and disease history, which may affect depression or depressive symptoms [7,13,18]. Physical injury was measured by a response of ‘yes’ to any of the following events that might have occurred in the participants’ families in the past 12 months: traffic accidents, tumbling, stinging by animals, cuts, scalds, and so on. For the question of disease history in the past, the participants were asked if they had the following diseases or symptoms: hypertension, diabetes, dyslipidemia, myocardial infarction, stroke, chronic obstructive pulmonary disease, asthma, cancer, and transient ischemic attacks.

### 2.5. Statistical Analysis

Notably, the scores of depressive symptoms were positively skewed, and there were many zero values in the data (66.0%), so we used the zero-inflated negative binomial (ZINB) regression to fit the data. Univariable and multivariable ZINB regression models were used to calculate the incidence rate ratios (IRRs) and 95% confidence intervals (CIs) for evaluating the potential association between SHS exposure and depressive symptoms. To explore the potential influences of covariates on the association between SHS exposure and depressive symptoms, two multivariable models were fitted. The multivariable model 1 (basic-adjusted model) was adjusted for sex, age, education, and per capita family income. The multivariable model 2 (fully adjusted model) was further adjusted for physical injury and number of disease history. Linear trends of SHS exposure were assessed by modeling exposure as continuous variables (arithmetic or logarithmic scale) in the ZINB models. To adjust for sampling design (clustering) in the analysis, the three-level nested model was fitted, but the intraclass correlations were not significant, so the fixed models were used in this study. A two-sided *p*-value of <0.05 was defined as being of statistical significance. All statistical analyses were performed using Stata version 14.0 (StataCorp LP, College Station, TX, USA).

## 3. Results

### 3.1. Characteristics of the Sample

As shown in Table 1, there was a total of 2771 non-smoking participants in this study, which is a representative sample of Guangdong no-smoking adults. As for the depressive symptoms, the scores ranged from 0 to 21, and the mean scores ± standard deviation were 1.1 ± 1.0, with significant differences according to sex (0.8 for males vs 1.2 for females, *p* < 0.001). The prevalence of SHS exposure in general was 33.5%, with no significant differences according to sex (32.2% for males vs 34.0% for females, *p* = 0.379). SHS exposure was the highest in restaurants (36.8%), followed by exposure in transport vehicles (23.0%), medical facilities (16.0%), and schools (12.0%). The mean age (± standard deviation) was 49.6 ± 14.0 years, 70.3% were female non-smokers; other sample characteristics are given in Table 1.

### 3.2. Association between SHS Exposure and Depressive Symptoms

The prevalence of depressive symptoms was significantly higher in participants with SHS exposure in general than in those without exposure (basic-adjusted model: IRR = 1.32, 95% CI 1.16–1.51; Table 2). When examining the relations by sources of exposure, there were similar positive associations for SHS exposure in medical facilities (basic-adjusted model: IRR = 1.37, 95% CI 1.17–1.61) and in schools (basic-adjusted model: IRR = 1.46, 95% CI 1.20–1.77), but a non-significant association was observed for SHS exposure in restaurants (basic-adjusted model: IRR = 0.95, 95% CI 0.82–1.09) and in public vehicles (basic-adjusted model: IRR = 1.11, 95% CI 0.95–1.31). As for continuous frequency of SHS exposure in general (days/week), there was a linear increasing dose-response relationship between SHS exposure and depressive symptoms (basic-adjusted model: IRR = 1.33, 95% CI 1.10–1.62), suggesting that the risk of depressive symptoms increased progressively as the days of SHS exposure increased. These results were unchanged in the fully adjusted models.

### 3.3. Association between SHS Exposure and Depressive Symptoms Stratified by Two Dimensions

The association between SHS exposure and cognitive–affective symptoms is showed in Table 3. Compared with no exposure, the participants with SHS exposure in general experienced a significantly higher prevalence rate of cognitive–affective symptoms (basic-adjusted model: IRR = 1.46, 95% CI 1.23–1.73). When examining the relationships by sources of exposure, there were similar positive associations for SHS exposure in medical facilities (basic-adjusted model: IRR = 1.52, 95% CI 1.23–1.87) and in schools (basic-adjusted model: IRR = 1.65, 95% CI 1.29–2.12). In addition, we observed a linear increasing dose-response relationship between continuous frequency of SHS exposure (days/week) and cognitive–affective symptoms (basic-adjusted model: IRR = 1.45, 95% CI 1.13–1.87).

The association between SHS exposure and somatic symptoms is also shown in Table 3. SHS exposure in general was positively associated with somatic symptoms (basic-adjusted model: IRR = 1.25, 95% CI 1.08–1.43), and similar positive associations were observed for SHS exposure in medical facilities (basic-adjusted model: IRR = 1.32, 95% CI 1.12–1.56) and in schools (basic-adjusted model: IRR = 1.30, 95% CI 1.06–1.60). In addition, we observed a linear increasing dose-response relationship between continuous frequency of SHS exposure (days/week) and somatic symptoms (basic-adjusted model: IRR = 1.29, 95% CI 1.05–1.58). These results were unchanged in the fully adjusted models for both cognitive–affective and somatic symptoms.

### 3.4. Association between SHS exposure and Depressive Symptoms Stratified by Sex

The association between SHS exposure and depressive symptoms for males and females is indicated in Table 4. For females, SHS exposure in general was positively associated with depressive symptoms (basic-adjusted model: IRR = 1.33, 95% CI 1.14–1.55), and a similar positive association was observed for SHS exposure in medical facilities (basic-adjusted model: IRR = 1.35, 95% CI 1.11–1.64) and in schools (basic-adjusted model: IRR = 1.41, 95% CI 1.11–1.79). In addition, we observed a linear increasing dose-response relationship between continuous frequency of SHS exposure (days/week) and depressive symptoms among females (basic-adjusted model: IRR = 1.33, 95% CI 1.07–1.66).

For males, people with SHS exposure in general demonstrated a significantly higher rate of depressive symptoms as compared with those without exposure (basic-adjusted model: IRR = 1.28, 95% CI 1.01–1.65), and a similar positive association was observed for SHS exposure in medical facilities (basic-adjusted model: IRR = 1.38, 95% CI 1.04–1.81) and in schools (basic-adjusted model: IRR = 1.43, 95% CI 1.07–1.90). However, in males, the association between continuous frequency of SHS exposure (days/week) and depressive symptoms was not statistically significant (basic-adjusted model: IRR = 1.32, 95% CI 0.87–1.99). These results were unchanged in the fully adjusted models for both females and males.

## 4. Discussion

This cross-sectional study revealed that non-smoking adults with setting-specific SHS exposure experienced a significantly higher prevalence of depressive symptoms than those without SHS exposure. The most striking finding from this study is that there was a monotonically increasing dose-response relationship between continuous frequency of SHS exposure and depressive symptoms. When differentiating this relationship by dimensions of depressive symptoms, there were similar dose-response relationships for cognitive-affective and somatic symptoms. When differentiating this relationship by sex, only females showed a significant dose-response relationship.

Much attention has been focused on the association between SHS exposure and the risk of depressive symptoms, but the findings are inconsistent [13,14,15,16,17,18]. Two studies on Korean non-smoking adults found that SHS exposure at home was associated with an increase in female depressive symptoms, but a non-significant association was found for SHS exposure in workplaces [15,16]. On the contrary, there was a non-significant association between SHS exposure at home and depressive symptoms among Japanese workers, but a significant association was observed for SHS exposure in workplaces [17]. In addition, in Chinese middle-aged women, positive associations for both home SHS exposure and workplace SHS exposure were found [18]. These above findings suggest a setting-specific relationship between SHS exposure and depressive symptoms. We noticed that most studies only focused on exploring this association for SHS exposure in homes or/and workplaces. Therefore, we differentiated the association in setting-specific (e.g., medical facilities, schools, restaurants, and public vehicles) SHS to make the exposure clearer. The present study found that there were positive associations for SHS exposure in medical facilities (IRR = 1.37, 95% CI 1.17–1.61) and in schools (IRR = 1.46, 95% CI 1.20–1.77), but no association was observed for SHS exposure in restaurants and in public vehicles. These findings suggest that the setting-specific associations may differ in different study participants, which may be due to differences in frequency and doses of SHS exposure in specific settings. These results also point out the urgent need for a comprehensive smoke-free legislation covering all public places in Guangdong to protect the public from SHS hazards. Although the biology and behavioral basis for smoking-attributable diseases has been explained beyond a shadow of a doubt [32], further research is needed to examine whether first- and secondhand smokers share similar mechanisms of association.

It remains unclear whether there is a dose-response relationship between SHS exposure and depressive symptoms. Some studies revealed linear increasing trends between frequency of SHS exposure and depressive symptoms among adolescents and pregnant women [33,34], but a non-significant trend was observed in a Korea study on adolescents [35], suggesting that these findings are inconsistent. The present study found a monotonically increasing dose-response relationship between continuous frequency of SHS exposure and depressive symptoms (IRR = 1.33, 95% CI 1.10–1.62), suggesting that the risk of depressive symptoms increased progressively as the days of SHS exposure increased. Compared with findings from binary SHS exposure, this finding of a dose-response relationship may provide more epidemiologic evidence to reveal the adverse effects of SHS exposure. Among Korean civilian women, a significant dose-response trend was only observed for home SHS exposure and not for workplace exposure [15]. On the contrary, among Japanese workers from small and medium-scale enterprises, the dose-response relationship was only observed for workplace SHS exposure and not for home exposure [17]. These findings provide more evidence for setting-specific dose-response relationships, which may be due to differences in geographical and environmental factors, study participants investigated, and levels of SHS exposure in their settings. Although no studies have evaluated the symptom-specific (cognitive-affective and somatic symptoms) association between SHS exposure and depressive symptoms, several studies have established differential associations between dimensions of depression and physical diseases (e.g., systemic sclerosis and diabetes), suggesting that the relationship between depression and chronic physical illness is stronger for the somatic symptoms [30,36]. The present study revealed that there were significant dose-response relationships for both cognitive–affective and somatic symptoms, indicating that the risk estimate for cognitive–affective symptoms is slightly higher than that for somatic symptoms. These findings highlight the need for further longitudinal studies to establish the causal relationship and the potential biological and behavioral mechanisms.

To date, it is still uncertain whether there is a sex-specific association between SHS exposure and depressive symptoms. Two Korean studies on non-smoking adults revealed that there was a significant dose-response pattern for household SHS exposure among women, but no significant association was observed for men [15,16]. These results support the hypothesis that women may be more susceptible to SHS-related factors. One possible explanation is that men’s higher rate of smoking contributes to women’s greater exposure to SHS in homes and workplaces [21,22]. In addition, various gender-related factors (such as gendered roles, unequal power differences between men and women, child-caring roles, and unequal earning power) affect women’s exposure to SHS and their capacity to control exposure, suggesting that a gender-sensitive framework is needed to develop research and tobacco control policies to address these issues [21]. Another possible explanation is that many women are exposed to SHS, especially in more stressful job or home environments. Women who reported severe stress levels were more likely to have greater SHS exposure at both home and workplace, indicating a positive relation between SHS exposure and stressful environments [15]. However, a national survey of Korean adolescents indicated that SHS exposure was positively associated with depression in both male and female adolescents [37]. Similarly, we found a significant association between SHS exposure in general (including SHS in medical facilities and SHS in schools) and depressive symptoms in both females and males. The above inconsistent results on sex-specific association may be due to differences in the prevalence of SHS exposure and depressive symptoms between females and males, measurement methods for SHS (self-report or plasma cotinine) and depressive symptoms (psychological scales or unstructured questions), populations investigated, and so on. 

There are potential biological and behavioral mechanisms that may explain the association between SHS exposure and depression. First, SHS exposure may be an indicator of stressful living and working environment, which contribute to the worsening of depressive symptoms through impaired neuroplasticity mechanisms [23]. Second, both chronic inflammation and neurobiological mechanism can elucidate this association [24,25,26]. Animal and human studies have indicated that SHS exposure is associated with adverse health effects, and in turn these adverse health effects may have direct and indirect effects on depression by chronic inflammation mechanism [24,25,26]. Other animal studies found that nicotine exposure may have long-term effects on the dopamine system and lead to long-term imbalance of dopamine transport, which may increase the risk for negative mood or depression [38,39].

A novel aspect of this study is the differentiation of the association in setting-specific exposure and symptom-specific outcomes, so as to make exposure and outcomes clearer. However, there are some potential limitations in this study. First, SHS exposure was evaluated by self-reports and not measured by biomarkers (e.g., serum nicotine and saliva nicotine). However, self-reported SHS exposure has been used in several population-based surveys and is generally valid [15,16]. Biochemical measures can give objective measurements but cannot distinguish the sources of exposure. Second, although depressive symptoms were not diagnosed by psychiatrists and relevant staffs, the PHQ-9 is a reliable screen for depression, because its reliability has been demonstrated in previous studies [40,41,42,43]. Third, the study was limited to a cross-sectional design; thus, we could only describe the association between SHS exposure and depressive symptoms and could not make a causal conclusion. This limitation can be overcome by future longitudinal studies. Finally, no SHS at home was obtained in the present study, so we could not determine the potential relationship between household SHS exposure and depressive symptoms.

## 5. Conclusions

In conclusion, this study found monotonically increasing dose-response relationships between frequency of SHS exposure and depressive symptoms among adults. In addition, we observed setting-specific and symptom-specific associations between SHS exposure and depressive symptoms. Future longitudinal studies are needed to establish the causal relationship and the biological mechanisms of the impact of SHS exposure.

## Figures and Tables

**Table 1:** Demographics characteristics of the non-smokers (*n* = 2771).

Characteristics	*n*	%
Sex		
Male	822	29.7
Female	1949	70.3
Age (years)		
18–24	90	3.2
25–34	304	11.0
35–44	638	23.0
45–54	739	26.7
≥55	1000	36.1
Education		
Primary school and below	1200	43.3
Junior high school	781	28.2
Senior high school	636	17.0
College and above	409	11.5
Per capita family income (¥)		
<3000	2216	78.0
3000–4000	285	10.3
>4000	270	9.7
Number of disease history		
0	2234	80.6
1	409	14.8
≥2	128	4.6
Physical injury		
No	3312	92.2
Yes	279	7.8
SHS exposure in general	927	33.5
SHS exposure in settings		
In medical facilities	444	16.0
In schools	331	12.0
In restaurants	1020	36.8
In transport vehicles	636	23.0

Abbreviations: *n*, number of participants; %, proportion of participants surveyed; SHS, secondhand smoke.

**Table 2:** Association between SHS exposure and depressive symptoms among non-smokers in Guangdong, China, 2010.

SHS Exposure	Univariable Model		Multivariable Model 1		Multivariable Model 2	
	IRR (95% CI)	*p*	IRR (95% CI) ^a^	*p*	IRR (95% CI) ^b^	*p*
SHS in general						
Frequency of SHS in general (days/week, logarithmic)	1.33 (1.08–1.64)	0.007	1.33 (1.10–1.62)	0.004	1.33 (1.10–1.62)	0.003
Binary SHS in general						
No	1.00		1.00		1.00	
Yes	1.27 (1.11–1.46)	0.001	1.32 (1.16–1.51)	<0.001	1.33 (1.17–1.52)	<0.001
SHS in settings						
SHS in medical facilities						
No	1.00		1.00		1.00	
Yes	1.31 (1.11–1.56)	0.002	1.37 (1.17–1.61)	<0.001	1.36 (1.17–1.60)	<0.001
SHS in schools						
No	1.00		1.00		1.00	
Yes	1.27 (1.05–1.55)	0.015	1.46 (1.20–1.77)	<0.001	1.51 (1.25–1.84)	<0.001
SHS in restaurants						
No	1.00		1.00		1.00	
Yes	0.91 (0.79–1.05)	0.206	0.95 (0.82–1.09)	0.446	0.94 (0.82–1.09)	0.413
SHS in public vehicles						
No	1.00		1.00		1.00	
Yes	1.13 (0.95–1.34)	0.157	1.11 (0.95–1.31)	0.182	1.14 (0.97–1.33)	0.111

Abbreviations: SHS, secondhand smoke; IRR, incidence rate ratio; CI, confidence interval. ^a^ Adjusted for sex, age, education, and per capita family income. ^b^ Adjusted for sex, age, education, per capita family income, physical injury, and number of disease history.

**Table 3:** Association between SHS exposure and depressive symptoms stratified by two dimensions.

SHS Exposure	Univariable Model		Multivariable Model 1		Multivariable Model 2	
	IRR (95% CI)	*p*	IRR (95% CI) ^a^	*p*	IRR (95% CI) ^b^	*p*
Cognitive–affective symptoms						
SHS in general						
Frequency of SHS in general (days/week, logarithmic)	1.51 (1.11–2.07)	0.009	1.45 (1.13–1.87)	0.004	1.45 (1.13–1.87)	0.004
Binary SHS in general						
No	1.00		1.00		1.00	
Yes	1.37 (1.11–1.69)	0.003	1.46 (1.23–1.73)	<0.001	1.48 (1.25–1.76)	<0.001
SHS in settings						
SHS in medical facilities						
No	1.00		1.00		1.00	
Yes	1.47 (1.14–1.89)	0.003	1.52 (1.23–1.87)	<0.001	1.53 (1.24–1.88)	<0.001
SHS in schools						
No	1.00		1.00		1.00	
Yes	1.47 (1.11–1.94)	0.007	1.65 (1.29–2.12)	<0.001	1.76 (1.37–2.26)	<0.001
SHS in restaurants						
No	1.00		1.00		1.00	
Yes	0.98 (0.79–1.22)	0.862	0.99 (0.82–1.19)	0.874	0.98 (0.82–1.18)	0.860
SHS in public vehicles						
No	1.00		1.00		1.00	
Yes	1.17 (0.92–1.50)	0.209	1.13 (0.92–1.38)	0.239	1.17 (0.95–1.43)	0.138
Somatic symptoms						
SHS in general						
Frequency of SHS in general (days/week, logarithmic)						
	1.22 (0.95–1.58)	0.125	1.29 (1.05–1.58)	0.015	1.29 (1.05–1.57)	0.015
Binary SHS in general						
No	1.00		1.00		1.00	
Yes	1.10 (0.93–1.31)	0.275	1.25 (1.08–1.43)	0.002	1.25 (1.09–1.43)	0.002
SHS in settings						
SHS in medical facilities						
No	1.00		1.00		1.00	
Yes	1.19 (0.97–1.47)	0.090	1.32 (1.12–1.56)	0.001	1.32 (1.12–1.56)	0.001
SHS in schools						
No	1.00		1.00		1.00	
Yes	1.08 (0.84–1.38)	0.555	1.30 (1.06–1.60)	0.012	1.35 (1.10–1.66)	0.004
SHS in restaurants						
No	1.00		1.00		1.00	
Yes	0.76 (0.63–0.91)	0.003	0.88 (0.76–1.03)	0.114	0.88 (0.75–1.02)	0.098
SHS in public vehicles						
No	1.00		1.00		1.00	
Yes	0.98 (0.79–1.21)	0.825	1.01 (0.85–1.19)	0.933	1.03 (0.87–1.22)	0.694

Abbreviations: SHS, secondhand smoke; IRR, incidence rate ratio; CI, confidence interval. ^a^ Adjusted for sex, age, education, and per capita family income. ^b^ Adjusted for sex, age, education, per capita family income, physical injury, and number of disease history.

**Table 4:** Association between SHS exposure and depressive symptoms among non-smokers stratified by sex.

SHS Exposure	Univariable Model		Multivariable Model 1		Multivariable Model 2	
	IRR (95% CI)	*p*	IRR (95% CI) ^a^	*p*	IRR (95% CI) ^b^	*p*
Females						
SHS in general						
Frequency of SHS in general (days/week, logarithmic)						
	1.32 (1.05–1.67)	0.020	1.33 (1.07–1.66)	0.011	1.36 (1.09–1.68)	0.006
Binary SHS in general						
No	1.00		1.00		1.00	
Yes	1.29 (1.09–1.51)	0.003	1.33 (1.14–1.55)	<0.001	1.36 (1.17–1.58)	<0.001
SHS in settings						
SHS in medical facilities						
No	1.00		1.00		1.00	
Yes	1.33 (1.08–1.63)	0.007	1.35 (1.11–1.64)	0.002	1.34 (1.11–1.62)	0.002
SHS in schools						
No	1.00		1.00		1.00	
Yes	1.31 (1.03–1.68)	0.030	1.41 (1.11–1.79)	0.004	1.50 (1.19–1.90)	0.001
SHS in restaurants						
No	1.00		1.00		1.00	
Yes	0.93 (0.78–1.10)	0.396	0.95 (0.80–1.13)	0.537	0.94 (0.79–1.12)	0.492
SHS in public vehicles						
No	1.00		1.00		1.00	
Yes	1.11 (0.90–1.36)	0.339	1.04 (0.86–1.27)	0.669	1.06 (0.88–1.29)	0.523
Males						
SHS in general						
Frequency of SHS in general (days/week, logarithmic)						
	1.23 (0.79–1.89)	0.357	1.32 (0.87–1.99)	0.191	1.31 (0.85–2.02)	0.222
Binary SHS in general						
No	1.00		1.00		1.00	
Yes	1.20 (0.93–1.55)	0.161	1.28 (1.01–1.65)	0.048	1.30 (1.01–1.69)	0.048
SHS in settings						
SHS in medical facilities						
No	1.00		1.00		1.00	
Yes	1.34 (1.01–1.79)	0.045	1.38 (1.04–1.81)	0.023	1.40 (1.05–1.86)	0.021
SHS in schools						
No	1.00		1.00		1.00	
Yes	1.32 (0.98–1.78)	0.066	1.43 (1.07–1.90)	0.016	1.47 (1.03–2.09)	0.033
SHS in restaurants						
No	1.00		1.00		1.00	
Yes	0.93 (0.72–1.20)	0.574	0.92 (0.71–1.19)	0.539	0.91 (0.70–1.18)	0.458
SHS in public vehicles						
No	1.00		1.00		1.00	
Yes	1.30 (0.99–1.70)	0.055	1.25 (0.95–1.64)	0.110	1.25 (0.95–1.65)	0.115

Abbreviations: SHS, secondhand smoke; IRR, incidence rate ratio; CI, confidence interval. ^a^ Adjusted for sex, age, education, and per capita family income. ^b^ Adjusted for sex, age, education, per capita family income, physical injury, and number of disease history.
